# Supplementary material for: Cdk1 Restrains NHEJ through Phosphorylation of XRCC4-like Factor Xlf1
Source: Cell Rep. 2014 Dec 18;9(6):2011–7. doi: 10.1016/j.celrep.2014.11.044 (PMC4542292; doi:10.1016/j.celrep.2014.11.044)
Supplement: Document S1. Supplemental Experimental Procedures, Figures S1–S3, and Table S1 [file mmc1.pdf]

Cell Reports, Volume *9*

Supplemental Information

Cdk1 Restrains NHEJ through Phosphorylation of XRCC4-like Factor Xlf1

Pierre Hentges, Helen Waller, Clara C. Reis, Miguel Godinho Ferreira, and Aidan J. Doherty

Supplemental Table 1: strains used

Name	genotype
PH111	xlf1:: natMX-nmt41-GFP-xlf1::loxP/M
PH113	xlf1:: natMX-nmt41-GFP-xlf1.T180A.S192A::loxP/M
PH369	cdc2.F84G xlf1:: natMX-nmt41-GFP-xlf1::loxP/M
PH371	cdc2.F84G xlf1:: natMX-nmt41-GFP-xlf1.T180A.S192A::loxP/M
PH365	cdc10.M17 xlf1:: natMX-nmt41-GFP-xlf1::loxP/M
PH360	cdc10.M17 xlf1:: natMX-nmt41-GFP-xlf1.T180A.S192A::loxP/M
PH001	ura4D-18 leu1-32 nmt41-xlf1::natMX
PH095	ade6-704 leu1-32 ura4-D18 xlf1::xlf1::loxP/M
PH097	ade6-704 leu1-32 ura4-D18 xlf1::xlf1. T180A.S192A::loxP/M
PH099	ade6-704 leu1-32 ura4-D18 xlf1::xlf1. T180E.S192E::loxP/M
MGF490	ade6-704 leu1-32 ura4-D18 taz1::natMX xlf1::ura4::loxP/M
MGF491	ade6-704 leu1-32 ura4-D18 xlf1::xlf1::loxP/M taz1::kan
MGF492	ade6-704 leu1-32 ura4-D18 xlf1.T180A.S192A::loxP/M taz1::kan
MGF493	ade6-704 leu1-32 ura4-D18 xlf1.T180E.S192E::loxP/M taz1::kan
MGF633	ade6-704 leu1-32 ura4-D18 xlf1.T180E.S192E::loxP/M xlf1EE ctp1::KanR taz1::URA4+
MGF669	leu1-32 ura4-D18 xlf1.T180A.S192A::loxP/M ctp1::KanR taz1::URA4+
MGF672	ura4D-18 leu1-32 nmt41-xlf1::natMX taz1::URA4+
MGF673	ura4D-18 leu1-32 nmt41-xlf1.AA::natMX taz1::URA4+
MGF1776	ade6-704 leu1-32 ura4-D18 xlf1.T180A.S192A::loxP/M taz1::kan lig4::natMX ctp1::hphMX

Table of the strains used and their genotypes. Related to Experimental Procedures.

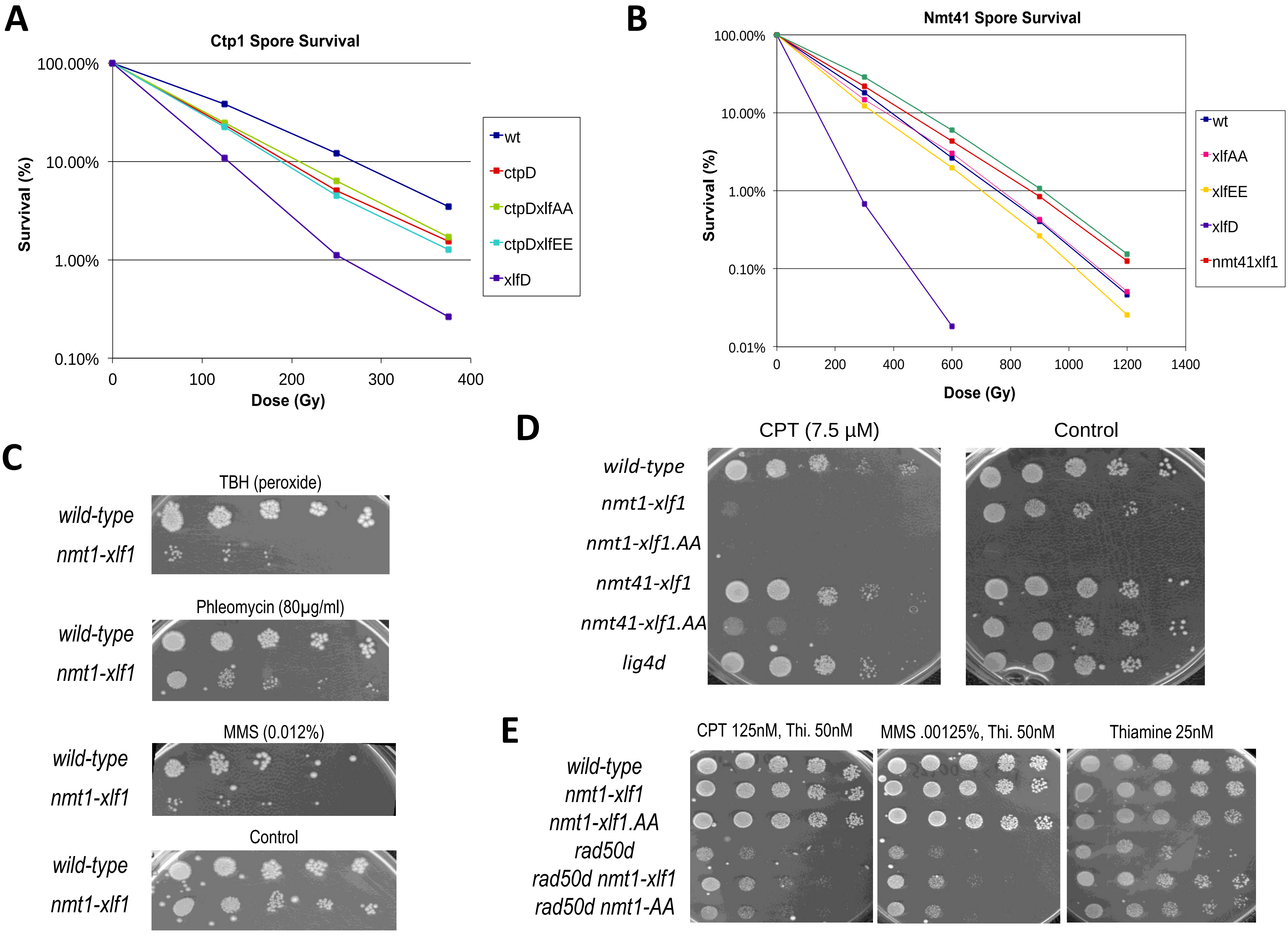
A
Ctp1 Spore Survival
100.00%
10.00%
1.00%
0.10%
Survival (%)
0
100
200
300
400
Dose (Gy)
wt
ctpD
ctpDxlfAA
ctpDxlfEE
xlfD
B
Nmt41 Spore Survival
100.00%
10.00%
1.00%
0.10%
0.01%
Survival (%)
0
200
400
600
800
1000
1200
1400
Dose (Gy)
wt
xlfAA
xlfEE
xlfD
nmt41xlf1
C
TBH (peroxide)
wild-type
nmt1-xlf1
Phleomycin (80µg/ml)
wild-type
nmt1-xlf1
MMS (0.012%)
wild-type
nmt1-xlf1
Control
wild-type
nmt1-xlf1
D
CPT (7.5 µM)
Control
wild-type
nmt1-xlf1
nmt1-xlf1.AA
nmt41-xlf1
nmt41-xlf1.AA
lig4d
E
CPT 125nM, Thi. 50nM
MMS .00125%, Thi. 50nM
Thiamine 25nM
wild-type
nmt1-xlf1
nmt1-xlf1.AA
rad50d
rad50d nmt1-xlf1
rad50d nmt1-AA

Figure S1

Supplemental Figure Legends

Figure S1 related to Figure 1

A. Spore survival of spores deleted for *ctp1*

B. Spore survival of *xlf1* point mutants

C. Spot tests with DNA damage treatment of strains overexpressing *xlf1* from medium-level *nmt41*

D. Spot tests with DNA damage treatment of *xlf1* mutants overexpressing from medium-level *nmt41* and high-level *nmt1*

E. Spot tests with DNA damage treatment of *xlf1* overexpression in *rad50* deletion background

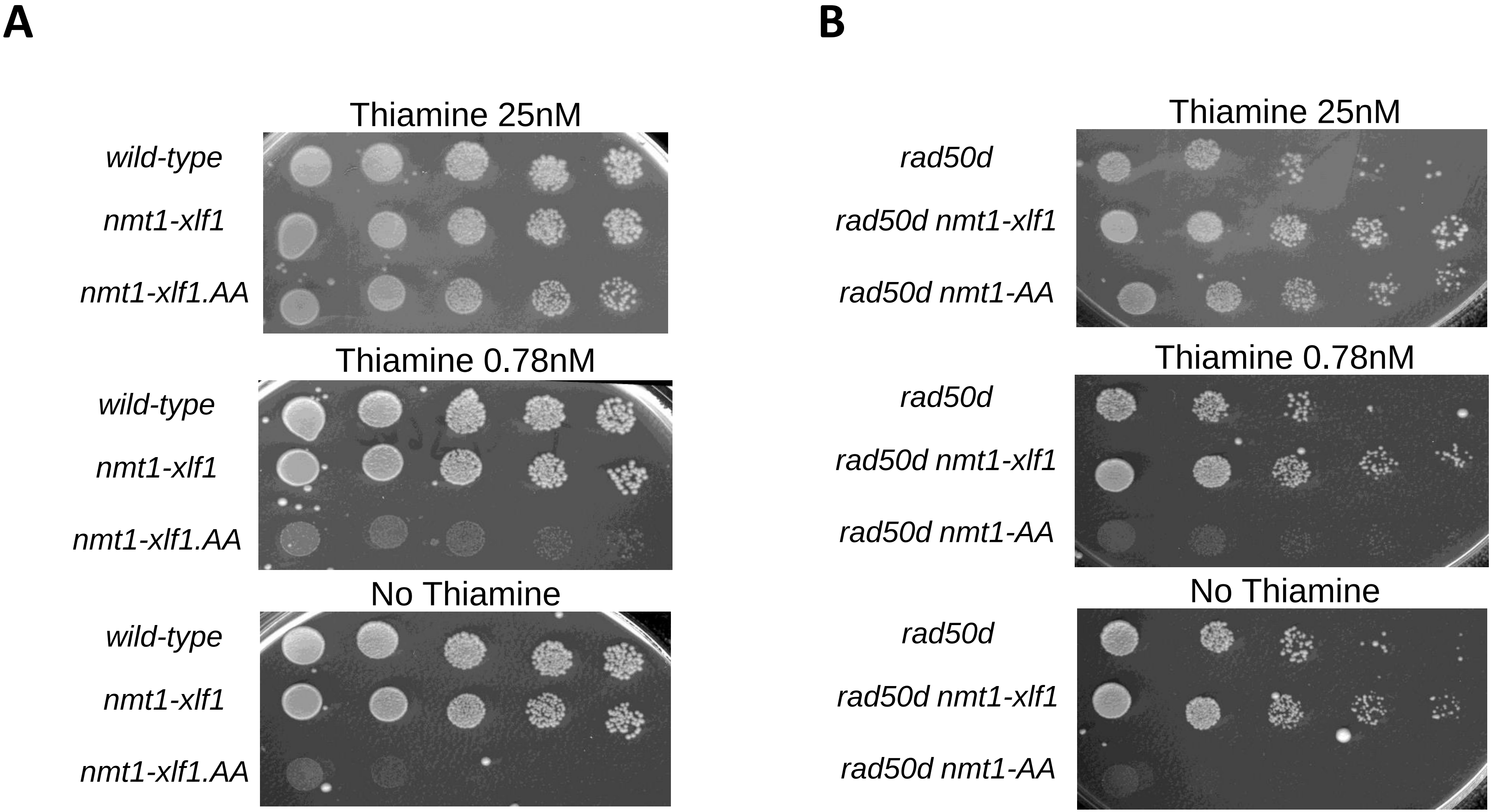
A
B
Thiamine 25nM
wild-type
nmt1-xlf1
nmt1-xlf1.AA
Thiamine 0.78nM
wild-type
nmt1-xlf1
nmt1-xlf1.AA
No Thiamine
wild-type
nmt1-xlf1
nmt1-xlf1.AA
Thiamine 25nM
rad50d
rad50d nmt1-xlf1
rad50d nmt1-AA
Thiamine 0.78nM
rad50d
rad50d nmt1-xlf1
rad50d nmt1-AA
No Thiamine
rad50d
rad50d nmt1-xlf1
rad50d nmt1-AA

Figure S2

Figure S2 related to Figure 2

A. Spot test with nmt1 high-level overexpression of *xlf1* mutants.

B. Spot test with nmt1 high-level overexpression of *xlf1* mutants in *rad50d* background.

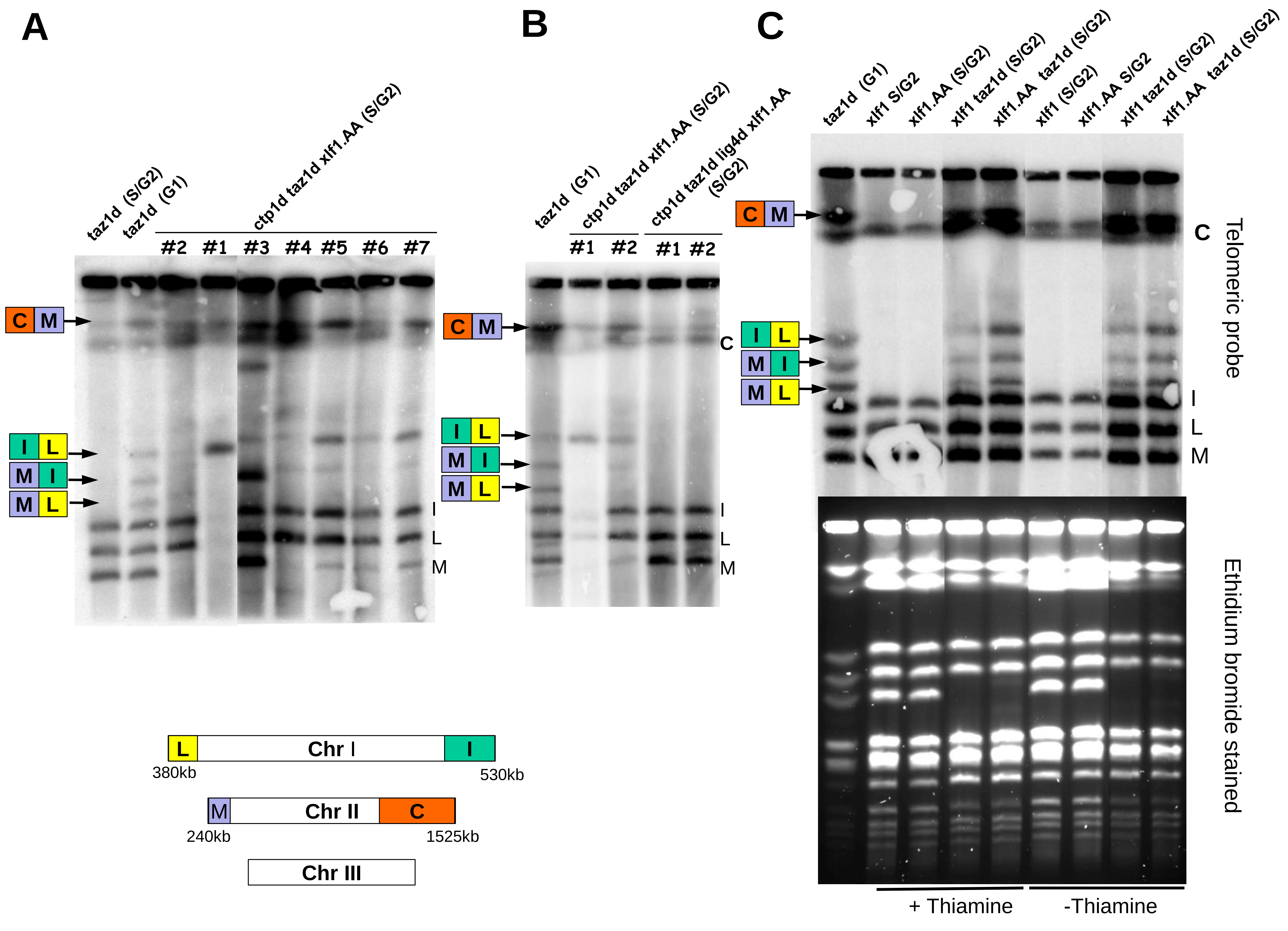
A
taz1d (S/G2)
taz1d (G1)
ctp1d taz1d xlf1.AA (S/G2)
#2 #1 #3 #4 #5 #6 #7
C M
I L
M I
M L
I
L
M
L
Chr I
I
380kb
530kb
M
Chr II
C
240kb
1525kb
Chr III
B
taz1d (G1)
ctp1d taz1d xlf1.AA (S/G2)
ctp1d taz1d lig4d xlf1.AA (S/G2)
#1 #2 #1 #2
C
C
taz1d (G1)
xlf1 S/G2
xlf1.AA (S/G2)
xlf1 taz1d (S/G2)
xlf1.AA taz1d (S/G2)
xlf1 (S/G2)
xlf1.AA S/G2
xlf1 taz1d (S/G2)
xlf1.AA taz1d (S/G2)
Telomeric probe
Ethidium bromide stained
+ Thiamine
-Thiamine

Figure S3

Figure S3 related to Figure 3

A. 7 independent clones were isolated by deleting *taz1* in *ctp1d xlf1.AA* cells through integration of a *kanMX* antibiotic resistance cassette. *xlf1.AA* is expressed at endogenous levels.

Diagram of telomeric NotI restriction fragments. Chromosomes I and II each release two telomeric restriction fragments (C, I, L and M). Chromosome III lacks NotI restriction sites; NotI digests of genomic DNA of the indicated strains were separated by PFGE and chromosomal end-to-end fusions were detected by Southern blot using a telomere probe (arrows indicate the positions of the resolved telomere fusions). Nitrogen starved *taz1d* was used as positive control for fusions.

B. NHEJ-mediated telomeric fusions in cycling cells of two independent clones of *ctp1d taz1d xlf1.AA* and two independent clones of *ctp1d taz1d lig4d xlf1.AA*. PFGE analysis and Southern blot using a using a telomere probe.

C. Southern blot (top) and ethidium bromide stained agarose gel (bottom) of PFGE shown in Fig 3. Decreases in the signal intensity from the telomere probe are not due to less DNA being loaded. Wild-type *xlf1* and *xlf1.AA* are expressed from the medium-strength thiamine-derepressible *nmt41* promoter.

Experimental Procedures

Genetic and Cell Studies

Media and standard genetic techniques were as described previously (Moreno et al., 1991). Spot tests and spore survival assays were carried out as in (Hentges et al., 2006) *xlf1* phosphorylation mutant strains were created by integrating DNA fragments containing T180A.S192A and T180E.S192E at the *xlf1* locus using a cre-lox method described in (Watson et al., 2008). *nmt41, nmt1* and *nmt41-GFP* strains were derived from this by integrating the corresponding cassettes described in (Van Driessche et al., 2005). Nat deletion strains were constructed as in (Hentges et al., 2005). For the *taz1d* assays, strains were created by integrating a kanMX cassette into the relevant background at the *taz1* locus while assays was carried out as described previously (Reis et al., 2012).

To determine the formation of Rad52 foci after IR, *nmt1-xlf1 rad52-GFP* and *nmt1-xlf1.AA rad52-GFP* cells were grown to log-phase in YNB without thiamine. A population of early G2 cells was isolated using centrifugation through a lactose gradient. Cells were treated with 50Gy gamma irradiation, attached to a concanavalin A-coated chamber. The presence of Rad52 foci was monitored over 4 hours.

DSB Plasmid Repair Assays

The plasmid religation assay using leucine selection (fig 2.a) was carried out as described previously (Manolis et al., 2001). The plasmid relegation assay using hygromycin selection (fig 2.b) was carried out in the same manner except that plasmid pRL1 bearing a hphMX marker was used, and that plasmid transformation was followed by a 2 hour incubation step before hygromycin selection was applied by plating cells on hygromcyin-containing media. The plasmid repair assay measuring HR and NHEJ in parallel (fig. 3.d) used plasmids pRL1 (hygromycin) for end-joining and pJK148 (leu1 marker) for chromosomal integration at *leu1*. $2x10^8$ mid-log phase cells were washed and incubated in 4ml DTT buffer for 15min at 30°C. Cells were washed twice with 2ml 1M sorbitol, resuspended in a total volume of 100μl by adding 70μl 1M sorbitol and then divided in two. For the NHEJ electroporations, 200ng uncut pAL19 and 600ng EcoRV and PvuII digested pRL1 plasmid. For the HR electroporations add 600ng uncut pRL1 and 400ng NdeI digested pJK148

plasmid DNA. Cells were then transferred to pre chilled electroporation cuvettes (0.2cm), electroporated at 1500V, 200Ω, 25μF. 950μL ice cold YNB with 1M sorbitol was added immediately and cells were incubated at 30°C for 2 hours. Cells were then plated on selective plates (YNB +ura +ade -leu, YEA +hyg) and incubated at 30°C for 4-5 days. NHEJ efficiency was calculated by dividing the number of hygromycin resistant colonies by the number of leucine positive colonies. HR efficiency was calculated by dividing leu+ colonies by the number of hygromycin resistant colonies. Values were normalised to wt = 100% for both repair pathways.

Protein methods

To separate and detect phosphorylated Xlf1 using the Phos-tag, samples were separated by SDS-PAGE on 12% gels containing 25µM Phos-tag (AAL-107 Wako) and 50µM $MnCl_2$. Prior to transfer onto PVDF, gels were incubated for 10min in transfer buffer with 1mM EDTA, then buffer without EDTA, followed by standard Western blotting. GFP-Xlf1 was detected using anti-GFP antibody from Invitrogen (1:2500 dilution).

For the experiment with *cdc10.M17* ts cells, cultures were grown to log-phase in EMM2 without thiamine at 25°C, blocked by growth at 36.5°C for one generation time, and released by switching temperature to 25°C. Whole cell lysates were prepared using the TCA method (Watson et al., 2008) and analysed by Western blotting with Phos-tag as described above.

Chk1 phosphorylation was analysed using lysates prepared using the TCA method, and detection of Chk1-HA using monoclonal anti-HA antibody (diluted 1:2000; Santa Cruz Biotechnology).

Recombinant Xlf1 protein with the mutations T180A, S192A, or T180A.S192A was prepared by site-directed mutagenesis of xlf1 on pET28a, followed by expression and purification as described previously (Hentges et al., 2006).

References

Van Driessche, B., Tafforeau, L., Hentges, P., Carr, A.M., and Vandenhaute, J. (2005). Additional vectors for PCR-based gene tagging in Saccharomyces cerevisiae and Schizosaccharomyces pombe using nourseothricin resistance. Yeast Chichester Engl. *22*, 1061–1068.

Hentges, P., Van Driessche, B., Tafforeau, L., Vandenhaute, J., and Carr, A.M. (2005). Three novel antibiotic marker cassettes for gene disruption and marker switching in Schizosaccharomyces pombe. Yeast Chichester Engl. *22*, 1013–1019.

Hentges, P., Ahnesorg, P., Pitcher, R.S., Bruce, C.K., Kysela, B., Green, A.J., Bianchi, J., Wilson, T.E., Jackson, S.P., and Doherty, A.J. (2006). Evolutionary and functional conservation of the DNA non-homologous end-joining protein, XLF/Cernunnos. J. Biol. Chem. *281*, 37517–37526.

Manolis, K.G., Nimmo, E.R., Hartsuiker, E., Carr, A.M., Jeggo, P.A., and Allshire, R.C. (2001). Novel functional requirements for non-homologous DNA end joining in Schizosaccharomyces pombe. EMBO J. *20*, 210–221.

Moreno, S., Klar, A., and Nurse, P. (1991). Molecular genetic analysis of fission yeast Schizosaccharomyces pombe. Methods Enzymol. *194*, 795–823.

Reis, C.C., Batista, S., and Ferreira, M.G. (2012). The fission yeast MRN complex tethers dysfunctional telomeres for NHEJ repair. EMBO J. *31*, 4576–4586.

Watson, A.T., Garcia, V., Bone, N., Carr, A.M., and Armstrong, J. (2008). Gene tagging and gene replacement using recombinase-mediated cassette exchange in Schizosaccharomyces pombe. Gene *407*, 63–74.
