# Supplementary material for: Cdk1 Restrains NHEJ through Phosphorylation of XRCC4-like Factor Xlf1
Source: Cell Rep. 2014 Dec 18;9(6):2011–7. doi: 10.1016/j.celrep.2014.11.044 (PMC4542292; doi:10.1016/j.celrep.2014.11.044)
Supplement: Document S2. Article plus Supplemental Information [file mmc2.pdf]

Cell Reports

Report

Cdk1 Restrains NHEJ through Phosphorylation of XRCC4-like Factor Xlf1

Graphical Abstract

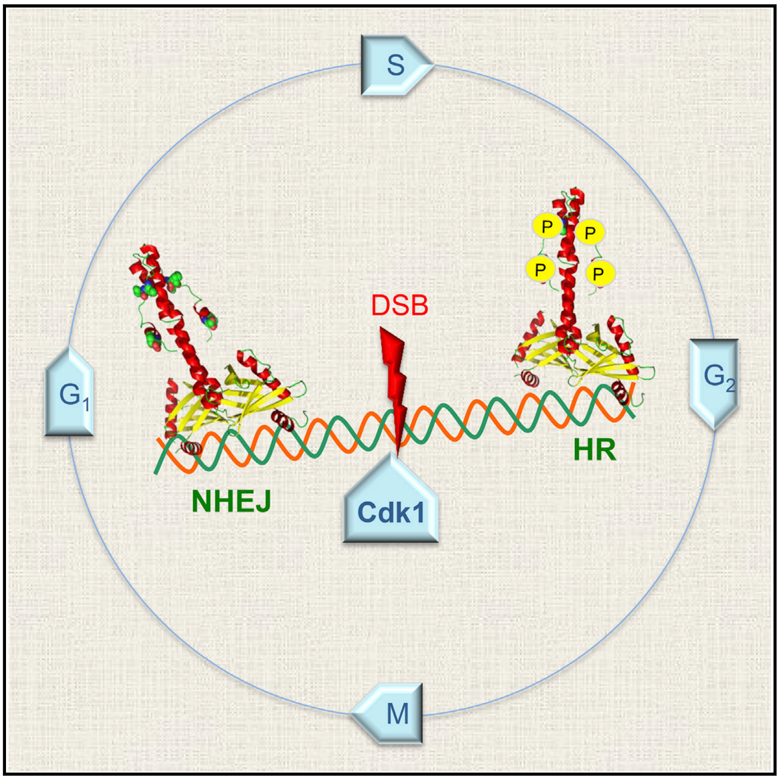

Highlights

- Cdc2[Cdk1] phosphorylates the core NHEJ factor Xlf1 in fission yeast
- Phosphorylation of Xlf1 inhibits nonhomologous end-joining (NHEJ)
- Cells with phospho-null Xlf1 have elevated levels of NHEJ repair
- NHEJ repair can predominate over HR when Cdc2[Cdk1] regulation of Xlf1 is lost

Authors

Pierre Hentges, Helen Waller, ..., Miguel Godinho Ferreira, Aidan J. Doherty

Correspondence

ajd21@sussex.ac.uk

In Brief

Repair of DNA double-strand breaks (DSBs) by homologous recombination is activated by the cell cycle kinase Cdk1. Hentges et al. now find that the NHEJ factor Xlf1 is phosphorylated by Cdk1 and that this modification restrains end-joining in cycling cells. Removal of this regulation alters DSB pathway selection in vivo.

CrossMark
Hentges et al., 2014, Cell Reports 9, 2011–2017
December 24, 2014 ©2014 The Authors
http://dx.doi.org/10.1016/j.celrep.2014.11.044

CellPress

Cell Reports
Report

OPEN ACCESS
CellPress

Cdk1 Restrains NHEJ through Phosphorylation of XRCC4-like Factor Xlf1

Pierre Hentges,[1] Helen Waller,[1] Clara C. Reis,[2] Miguel Godinho Ferreira,[2] and Aidan J. Doherty[1,*]
[1]Genome Damage and Stability Centre, School of Life Sciences, University of Sussex, Brighton BN1 9RQ, UK
[2]Instituto Gulbenkian de Ciência, Oeiras 2781-901, Portugal
*Correspondence: ajd21@sussex.ac.uk
http://dx.doi.org/10.1016/j.celrep.2014.11.044
This is an open access article under the CC BY license (http://creativecommons.org/licenses/by/3.0/).

SUMMARY

Eukaryotic cells use two principal mechanisms for repairing DNA double-strand breaks (DSBs): homologous recombination (HR) and nonhomologous end-joining (NHEJ). DSB repair pathway choice is strongly regulated during the cell cycle. Cyclin-dependent kinase 1 (Cdk1) activates HR by phosphorylation of key recombination factors. However, a mechanism for regulating the NHEJ pathway has not been established. Here, we report that Xlf1, a fission yeast XLF ortholog, is a key regulator of NHEJ activity in the cell cycle. We show that Cdk1 phosphorylates residues in the C terminus of Xlf1 over the course of the cell cycle. Mutation of these residues leads to the loss of Cdk1 phosphorylation, resulting in elevated levels of NHEJ repair in vivo. Together, these data establish that Xlf1 phosphorylation by Cdc2^{Cdk1} provides a molecular mechanism for downregulation of NHEJ in fission yeast and indicates that XLF is a key regulator of end-joining processes in eukaryotic organisms.

INTRODUCTION

The ability to repair DNA damage is critically important for the preservation of genomic integrity. DNA double-strand breaks (DSBs) can be repaired by two different cellular pathways: homologous recombination (HR) and nonhomologous end-joining (NHEJ) (Symington and Gautier, 2011). HR processes use undamaged homologous DNA sequences—typically from the sister chromatid—as a repair template, thus enabling error-free repair. NHEJ can also restore chromosome integrity by religation of DSB ends (Chiruvella et al., 2013) in the absence of homologous sequences but is potentially more error prone. While core factors such as Ku, XRCC4, XRCC4-like Factor (XLF), and DNA ligase 4 are required for all NHEJ repair reactions, accessory factors, including polymerases and nucleases, are also needed to process termini of imprecise DSBs into ligatable substrates.

The relative preference for break repair pathways differs between eukaryotes. Mammalian cells use NHEJ as the predominant DSB repair mechanism, where the pathway is available throughout the cell cycle. Yeast prefer to repair DSBs by HR (Manolis et al., 2001). Nevertheless, most eukaryotes utilize both NHEJ and HR; therefore, the choice of repair pathway is crucial for cell survival. DSB repair pathway selection is regulated in the cell cycle, with NHEJ predominating in G_1 phase and HR restricted to the G_2 and S phases of the cell cycle (Ferretti et al., 2013). Cyclin-dependent kinase 1 (Cdk1) plays a key role in regulating end resection during HR. Resection is strongly inhibited by low Cdk1 activity in G_1 and can be reduced in G_2 by Cdk1 inhibition (Aylon et al., 2004; Ira et al., 2004). In mammalian cells and budding yeast, the main target of CDK phosphorylation is CtIP/Sae2, which facilitates DSB end resection (Huertas et al., 2008). Cdk1 phosphorylation also influences later steps in HR, as well as expression levels of HR proteins.

In budding yeast, the initiation of DSB resection is normally suppressed in G_1 due to low Cdk1 activity and depends on the MRX complex (Clerici et al., 2008). However, this dependence on Cdk1 activity can be overcome by deletion of Ku, suggesting that it induces indirect control over NHEJ by affecting HR instead. Several potential Cdk1 phosphorylation sites have been found in budding yeast Ku70/Ku80; however, their mutation did not affect NHEJ activity (Zhang et al., 2009). Thus, direct Cdk1 targets for NHEJ regulation have not yet been identified. In fission yeast, there is a reciprocal relationship between the deployment of the two major DSB pathways with NHEJ functioning during G_1 and HR predominant in G_2 cells (Ferreira and Cooper, 2004). It has been proposed that Cdk1 may influence this pathway selection, but a mechanism has not been identified.

Xlf1 is the fission yeast homolog of XLF/Cernunnos (Hentges et al., 2006; Cavero et al., 2007), a core NHEJ factor that binds to DNA and stimulates end-joining. In the present study, we identify Xlf1 as a key regulator of NHEJ activity in the cell cycle. We report that Cdk1 phosphorylates specific residues in the C terminus of Xlf1 over the course of the cell cycle. Using phospho-null and phosphomimic mutant strains, we demonstrate that Xlf1 phosphorylation inhibits the NHEJ pathway. We also identify effects on the checkpoint response and cellular events related to DSB resection. Together, these data establish that Xlf1 phosphorylation by Cdc2^{Cdk1} provides a molecular mechanism for the downregulation of NHEJ in fission yeast and offers insights into how this pathway may be regulated in other eukaryotic organisms.

RESULTS AND DISCUSSION

Cdk1 Phosphorylates Xlf1 In Vitro

NHEJ is tightly regulated in fission yeast, but the mechanism is unknown (Ferreira and Cooper, 2004). To identify if posttranslational

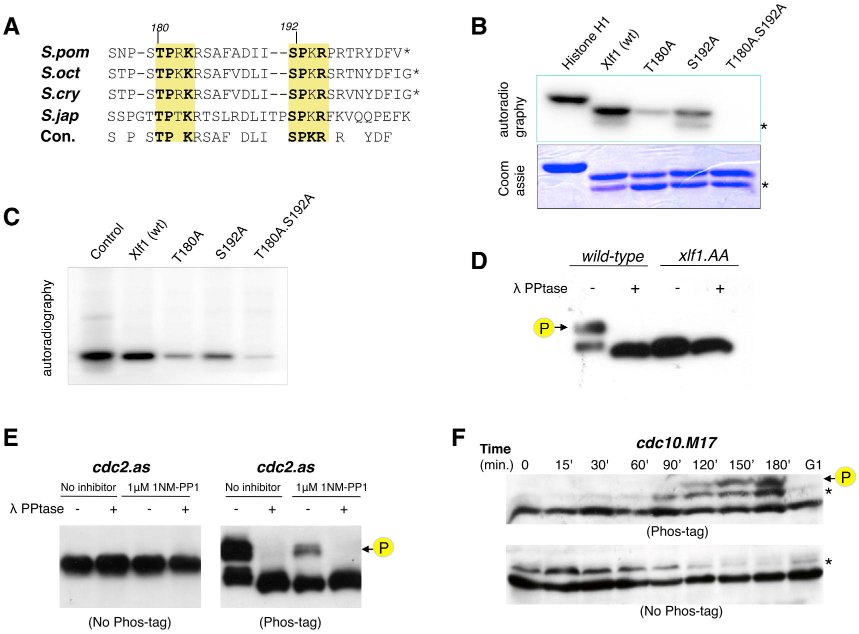

Figure 1. Xlf1 Is Phosphorylated by Cdk1

(A) Alignment of the C-terminal portion of four fission yeast *xlf1* homologs (*S. pombe*, *S. octosporus*, *S. cryophilus*, and *S. japonicus*), displaying two conserved cdc2[cdk1] phosphorylation motifs (yellow boxes): [ST]Px[KR].

(B) In vitro Cdk1 kinase assay. Recombinant Xlf1 protein was incubated with mammalian Cdk1 in the presence of γ-^{32}P-ATP and analyzed by autoradiography. Proteins used were the Cdk1 substrate histone H1 (positive control, 32 kDa), wild-type Xlf1 (27 kDa), single mutations of T180A and S192A, and double mutations T180A.S192A (AA). Recombinant Xlf1 is susceptible to cleavage between T180 and S192, and the cleavage product (indicated by an asterisk) is visible in the Coomassie-stained loading control.

(C) In vitro kinase assay using *S. pombe* Cdc2. Kinase assay with recombinant protein was conducted as in (B), except using Cdc2 complex purified from *S. pombe* cells.

(D) In vivo phosphorylation of Xlf1. GFP-tagged Xlf1 was immunoprecipitated from cell extracts of wild-type and *xlf1.T180A.S192A* (*xlf1.AA*), treated or mock treated with lambda phosphatase, and separated by SDS-PAGE in the presence of the phosphate-binding retardant Phos-tag. Phosphorylated Xlf1 is indicated by an arrow.

(E) GFP-tagged wild-type Xlf1 was immunoprecipated from cells containing the Shokat active site mutation cdc2.F84G (Dischinger et al., 2008) that had either been treated or mock treated with the inhibitor 1NM-PP1. Immunoprecipitates were treated or mock treated with lambda phosphatase and analyzed by SDS-PAGE and immunoblotting in either the presence or the absence of Phos-tag.

(F) A temperature-sensitive mutation was used to block *cdc10-M17 nmt41-GFP.xlf1* cells in G_1 phase and then released into the cell cycle. Phosphorylation of GFP-Xlf1 was analyzed by SDS-PAGE and immunoblotting of cell extracts in the presence or absence of Phos-tag. A nonspecific band detected by the GFP antibody is indicated by an asterisk.

modifications regulate NHEJ in *S. pombe*, we analyzed the sequences of the core factors (Ku, Lig4, and Xlf1). The C-terminal region of Xlf1 has two sites, T180 and S192, that conform to [ST]-P-x-[KR], a consensus motif for phosphorylation by the Cdc2[Cdk1] kinase. These potential phosphorylation sites are conserved in other fission yeasts (Figure 1A). To test if these sites serve as substrates for Cdc2[Cdk1], we mutated them to alanine and performed kinase assays. Wild-type (WT) and mutated Xlf1 proteins were incubated with mammalian Cdk1 kinase (Figure 1B) or *S. pombe* Cdc2[Cdk1] complex (Figure 1C). Similarly to histone H1, a known Cdc2[Cdk1] substrate (Moreno et al., 1989), Xlf1 was phosphorylated, establishing it as an in vitro substrate for the mammalian and the fission yeast kinase. Phosphorylation of single point mutants (Xlf1.T180A or Xlf1.S192A) was markedly reduced compared to WT Xlf1, suggesting that the two sites can be phosphorylated. When both residues were mutated to alanine (Xlf1.T180A.S192A or Xlf1.AA), Xlf1 phosphorylation was abolished. Thus, Cdc2[Cdk1] can phosphorylate Xlf1 on two conserved C-terminal residues in vitro.

Xlf1 Is Phosphorylated by Cdk1 in a Cell-Cycle-Dependent Manner

Next, we sought to investigate if phosphorylation of Xlf1 occurs in vivo. While phosphorylation had no apparent effect on the migration of Xlf1 in standard SDS-PAGE (Figures 1B and 1E), addition of a phosphate-binding agent, Phos-tag (Kinoshita et al., 2006), resolved phosphorylated Xlf1 as a distinct band. GFP-tagged Xlf1 was immunoprecipitated from asynchronous cultures and analyzed by Phos-tag western blotting. The slower migrating species was abolished by treatment with lambda phosphatase (Figure 1D), confirming that this represented phosphorylated cellular Xlf1. This species was absent in *xlf1.AA*, a strain that expresses Xlf1.AA, regardless of treatment with phosphatase. Together, these results demonstrate that Xlf1 is phosphorylated at T180 and S192 in vivo and indicate that further Cdc2[Cdk1] kinase sites are unlikely.

To determine if Cdc2[Cdk1] is responsible for Xlf1 phosphorylation in vivo, we used a *cdc2-as* mutant strain in which we could inhibit Cdc2[Cdk1] activity (Dischinger et al., 2008). Treatment of *cdc2as GFP-xlf1* cultures with the inhibitor (1NM-PP1) caused a significant reduction of the phosphoband of Xlf1 in Phos-tag western blots, which could be further reduced by phosphatase treatment (Figure 1E). This establishes that Xlf1 is phosphorylated by Cdc2[Cdk1] in unperturbed asynchronous cultures.

Cdc2[Cdk1] activity increases from a minimum in G_1 to levels peaking in G_2, triggering entry into mitosis. To study changes in Xlf1 phosphorylation status as cells progressed through the cell cycle, we used a temperature-sensitive *cdc10* mutant (*cdc10-M17*) to synchronize GFP-*xlf1* cultures in G_1. A phospho-Xlf1 species was not detectable in G_1-arrested cells (Figure 1F); however, upon release from the arrest, phosphorylated Xlf1 appeared and increased after 120 min, as cells entered G_2 phase. These data indicate that Cdc2[Cdk1] phosphorylates Xlf1 in a cell-cycle-dependent manner.

OPEN
ACCESS
CellPress

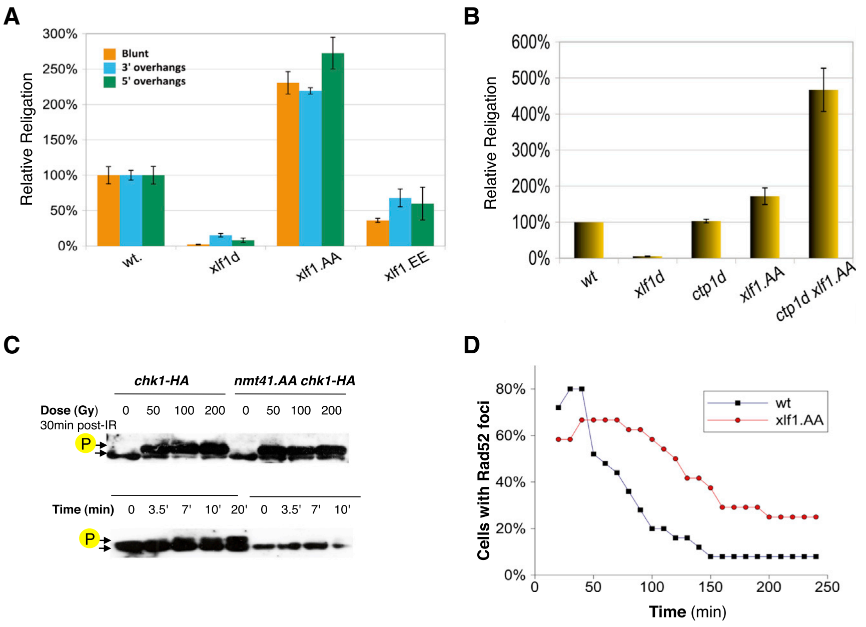

Figure 2. Phospho-Null *xlf1* Increases DNA End-Ligation Activity and Attenuates Damage Responses

(A) Religation of linearized plasmids containing blunt ends, 3′ and 5′ overhangs. Leu+ selection used to monitor religated plasmids. Data are reported as the mean ± 95% confidence interval.

(B) Religation of linearized plasmids with hygromycin selection within 2 hr of electroporation. Data are reported as the mean ± 95% confidence interval.

(C) Chk1-HA phosphorylation in response to ionizing radiation was monitored in wild-type and *nmt41-xlf1.AA* cells. Cells irradiated with 50 Gy, 100 Gy, and 200 Gy were analyzed 30 min after irradiation. To characterize the kinetics of the checkpoint response, cells irradiated with 50 Gy were also analyzed at 3.5 min, 7 min, and 10 min after irradiation. Phosphorylated Chk1 is indicated.

(D) The formation and persistence of Rad52-GFP foci following 50 Gy irradiation were monitored in wild-type and *nmt41-xlf1.AA* cells.

Xlf1 Phosphorylation by Cdc2^{Cdk1} Alters the Repair of DSBs by NHEJ

As NHEJ is most active in G_1 but inhibited in S/G_2 (Ferreira and Cooper, 2004), we predicted that Xlf1 phosphorylation inhibits NHEJ. To test this hypothesis, we examined the ability of *xlf1* phosphorylation mutants to religate linearized plasmid DNA (Manolis et al., 2001). Leucine auxotrophic cells were transformed with linearized plasmid DNA containing the *LEU2* marker. Their ability to religate ends and form colonies on selective plates lacking leucine was assessed by comparison to cells transformed with uncut plasmid. Log-phase cultures in which Cdc2^{Cdk1} phosphorylation of Xlf1 had been abolished (*xlf1.AA′*) displayed a ~2.5-fold increase in end-joining compared to WT cells (Figure 2A). In contrast, the Xlf1 phosphomimetic mutant *xlf1.EE* showed a moderate decrease in plasmid end-joining. The religation levels were unaffected by the nature of the ends (blunt or overhangs) in *xlf1* mutants. These results indicated that an inability to phosphorylate Xlf1 leads to increased NHEJ activity and supports the hypothesis that Cdc2^{Cdk1} phosphorylation of Xlf1 inhibits NHEJ.

The resection of DSBs is thought to make them unsuitable for end-joining. Therefore, we asked if we could increase levels of end-joining of linearized plasmids by impairing the resection of DNA ends. We repeated the plasmid religation assays in a strain lacking the resection gene *ctp1*$^+$. Deleting *ctp1* had no effect on plasmid religation levels (Figure 2B), suggesting that preventing Ctp1-dependent resection did not channel plasmid DSBs into NHEJ. However, when *ctp1*$^+$ was deleted in *xlf1.AA* cells, end-joining levels increased >4-fold compared to WT levels, significantly above an ≈2-fold increase caused by *xlf1.AA* in the presence of functional Ctp1. This finding suggested that, in log-phase cells, Ctp1 and phosphorylated Xlf1 synergistically counteract end-joining of linearized plasmid DNA.

Xlf1.AA Slows Down Cellular Responses to DSBs

Next, we characterized cellular response to ionizing radiation (IR)-induced DSBs in Xlf1 phospho-null strains. A key event in the cellular response to DNA damage is the activation of DNA damage checkpoints that arrest progress in the cell cycle to ensure that DSBs can be repaired. We monitored the phosphorylation status of Chk1 kinase, a marker of G_2 checkpoint activation (Walworth and Bernards, 1996), in response to IR. IR-induced Chk1 phosphorylation was observed in *nmt41-xlf1.AA chk1-HA* cultures at all IR doses, with no discernible difference to WT *chk1-HA* cultures (Figure 2C). We also characterized the kinetics of the checkpoint response. In *chk1-HA* cells, Chk1 phosphorylation reached a maximum value within 10 min. In contrast, the phospho-Chk1 band did not appear until 10 min after irradiation, rising further at later time points. This observation suggests that nonphosphorylatable Xlf1 causes a deceleration of the checkpoint response, though full induction of the G_2 checkpoint, as measured by Chk1 phosphorylation, is still achieved within 30 min.

Next, we studied the processing of IR-induced DSBs. Rad52 foci form after strand resection has begun and channeled into recombination processes (Symington and Gautier, 2011). In order to study the possible interference of phospho-null Xlf1 on such processes, we analyzed Rad52-GFP foci formed in live cells following IR. We irradiated *rad52-GFP* and *nmt41-xlf1.AA rad52-GFP* cells with 50 Gy and monitored the formation and persistence of Rad52-GFP foci using live-cell imaging. While Rad52 foci were observed in most cells with both WT *xlf1* and *nmt41-xlf1.AA*, foci formation was slower in *nmt41-xlf1.AA* (Figure 2D). Rad52 foci formation peaked ~30 min after IR in WT cells but 40–70 min after IR in *nmt41*-xlf1.AA, in which Rad52 foci also persisted for longer. This observation is consistent with HR processes, including DSB resection, being slowed in *nmt41-xlf1.AA* cells. We did not observe a general decrease of Rad52 foci in *nmt41-xlf1.AA*. As

Rad52 foci are associated with resection, and as resected DSBs are not a suitable substrate for NHEJ, the observed deceleration of HR is unlikely to be the result of the overall balance of DSB repair pathways being tipped in favor of NHEJ.

Overexpression of Xlf1.AA Sensitizes Cells to DNA Damage

As the balance between HR and NHEJ is tightly regulated in the cell cycle, we next asked if deregulation of repair pathways resulted in altered sensitivity to DNA damage. To address this question, we characterized the IR sensitivity of Xlf1 phosphomutants in a stationary state, not requiring cell cycle regulation. Spores from homozygous crosses of *xlf1.AA* displayed an IR sensitivity similar to that of WT spores, in contrast to a small but reproducible increase in IR sensitivity with *xlf1.EE* spores (Figure S1). This suggests that NHEJ is active but can be attenuated, as observed in the phosphomimetic *xlf1* mutant. Similarly, while deletion of *ctp1* increased IR sensitivity of spores, there was a small but reproducible increase in radioresistance in *xlf1.AA ctp1d*, whereas the opposite effect was evident with *xlf1.EE ctp1d* spores (Figure S1). The IR sensitivity of *ctp1d* spores may be due to damage other than DSBs induced in the spore state, such as single-strand breaks and other lesions impeding the first round of replication following germination. Overexpression of WT *xlf1* and *xlf1.AA* from *nmt41* increased the IR resistance in spores above WT levels. This is again compatible with the notion that Xlf1 is a limiting factor regulating NHEJ levels.

In contrast to spores, mutation of the Xlf1 phosphorylation sites did not have a discernible effect on the sensitivity of vegetative cells to a range of DNA damage treatments (data not shown). However, overexpression using the medium-level *nmt41* promoter of Xlf1 in vegetative cells rendered *nmt41-xlf1.AA* cells mildly sensitive to a variety of DNA-damaging agents, such as camptothecin, *tert*-butyl hydroperoxide, phleomycin, and methyl methanesulfonate in comparison to *nmt1-xlf1* (WT) (Figure S1). The sensitivity was increased in *nm41-xlf1.AA*. No increase in *xlf1.AA* DNA damage sensitivity was observed in strains in which *rad50* had been deleted (Figure S1), revealing an epistatic relationship with *rad50*. In addition, we noted that high-level overexpression from the *nmt1* promoter of *xlf1.AA*, but not with WT *xlf1*, caused cells to become inviable (Figures S1 and S2).

NHEJ Is Hyperactivated in an Xlf1 Cdk1 Phosphorylation Null Mutant

As plasmid religation assays have relaxed requirements for NHEJ (Almeida and Godinho Ferreira, 2013), we sought another in vivo assay to study the role of Xlf1 phosphorylation by Cdc2^{Cdk1} on chromosomal DSBs. Chromosome end fusions can be generated by NHEJ in cells with unprotected telomeres, such as *taz1d* (Ferreira and Cooper, 2004). However, chromosome end fusions are only generated if *taz1d* cells are arrested in G_1 but not during S/G_2, because of the downregulation of NHEJ activity in S/G_2 phases. If cell cycle inhibition of NHEJ activity restricts *taz1d* chromosome fusions to G_1 phase, disinhibition of NHEJ should lead to chromosome fusions in S/G_2-phase *taz1d* cells. Therefore, *taz1d* strains provide a suitable assay to study the potential inhibitory effect of Xlf1 phosphorylation on NHEJ in vivo. We first compared G_1-arrested (nitrogen-starved) and S/G_2 (log-phase) cultures in which telomeres were unprotected due to *taz1*$^+$ deletion. Chromosome fusions were detected in *taz1d* cells in G_1 but not in S/G_2 cells (Figure 3A). In contrast, no fusions appeared in G_1-arrested *taz1d xlf1d* cells, because of downregulation of NHEJ, as expected. Chromosome fusions were also observed in *taz1d xlf1.AA* cells when arrested in G_1 phase, showing that mutation of the phosphorylation sites preserves the ability to carry out NHEJ. It is important to note, however, that fusions were not detected in *taz1d xlf1.AA* during S/G2 phases. Thus, contrary to our prediction, disinhibition of NHEJ by preventing Xlf1 phosphorylation is not sufficient to fuse unprotected chromosome ends in log-phase cultures.

NHEJ and HR are regulated independently in the fission yeast cell cycle, as inactivation of HR does not lead to increased use of NHEJ in G_2 cells (Ferreira and Cooper, 2004). Conversely, we expected that abnormal activation of NHEJ in G_2 cells will take place in the presence of activated HR, since the disinhibition of NHEJ alone in the *xlf1.AA* mutant would not affect HR activity. Therefore, we reasoned that competition for DSBs between NHEJ and HR is likely taking place in *taz1d xlf1.AA* cells, potentially masking the disinhibition of NHEJ, which may only become detectable once HR is inactivated. To test this hypothesis, we repeated the experiment in a background in which *ctp1* was deleted. Ctp1 is essential for the initiation of resection, targeting DSBs toward HR (Limbo et al., 2007). When the chromosome fusion assays were repeated, no chromosome fusions were detected in *ctp1d taz1d xlf1*$^+$ during S/G_2 phases (Figure 3B), establishing that inactivation of Ctp1-dependent resection alone is not sufficient to cause fusion of unprotected chromosome ends. Strikingly, inactivation of HR in *ctp1d taz1d xlf1.AA* cells led to substantial telomere fusions. We analyzed seven independent clones (Figure S3A) and found that all contained intra- and/or interchromosomal end fusions, including clones with circular chromosomes (both termini of a chromosome are fused) during S/G_2 phases, a striking effect of NHEJ activity as confirmed by its absence in the corresponding *lig4d* strain (Figure S3B). This indicates that *xlf1.AA* prevents the inhibition of NHEJ in G_2 cells when HR is inactivated. In contrast, no chromosome fusions were detected in the corresponding phosphomimicking *xlf1* mutant, *ctp1d taz1d xlf1.EE*. These observations support the hypothesis that Cdc2^{Cdk1} phosphorylation of Xlf1 switches off NHEJ during the cell cycle.

To further explore the nature of competition between HR and NHEJ when both DSB repair pathways are active in the *xlf1.AA* phospho-null mutant, we speculated that overexpression of *xlf1*$^+$ may overcome the requirement to inactivate HR. We constructed *taz1d* strains in which *xlf1*$^+$ is controlled by the medium-strength *nmt41* promoter integrated at the *xlf1*$^+$ locus. Chromosome fusions could be detected in log-phase *nmt41-xlf1.AA taz1d* cells, even though HR was functional (Figure 3C). Overexpression of WT *xlf1*$^+$ similarly caused telomere fusions in the presence of HR function, though to a lesser extent than in the phospho-null mutant. This suggests that *xlf1*$^+$ overexpression contributes to overcoming recombinogenic mechanisms that prevent chromosome fusions, tipping the HR/NHEJ balance

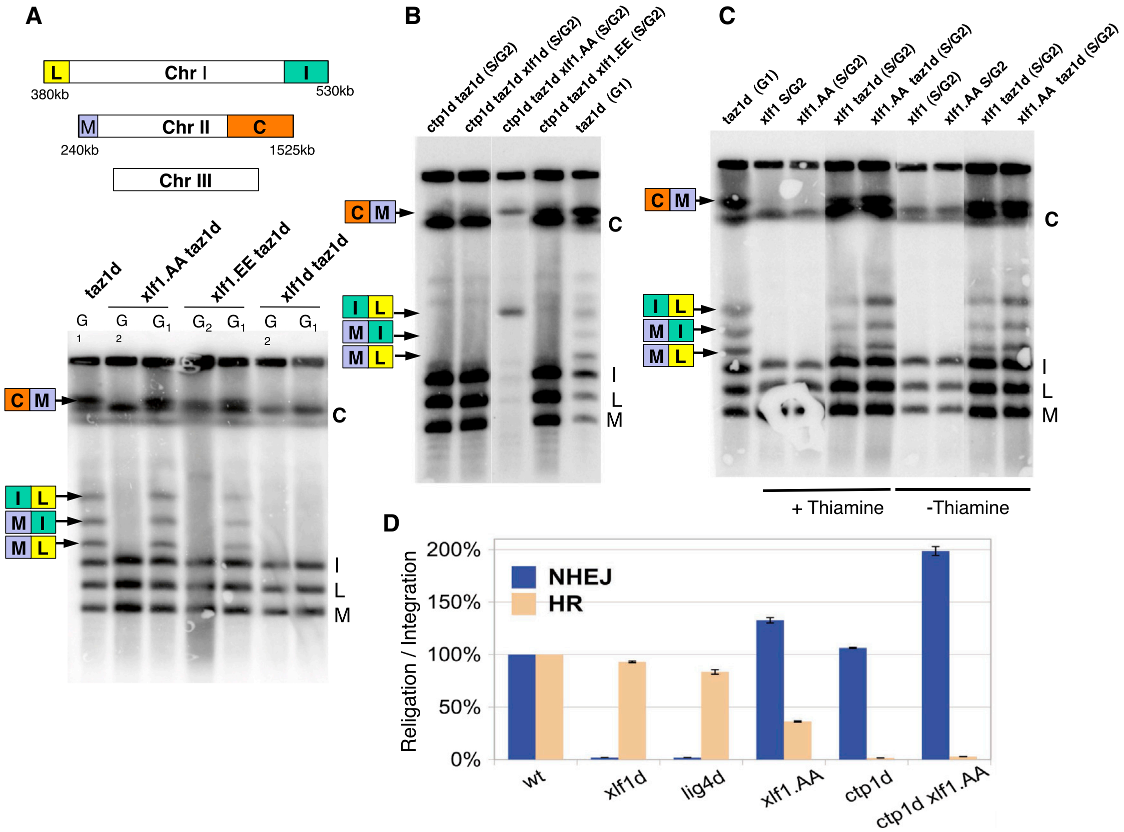

Figure 3. ***xlf1.AA*** **Promotes NHEJ Fusion of Unprotected Telomeres**

(A) Ligation of chromosome ends in *xlf1* mutants in *taz1d* strains. Scheme of telomeric NotI restriction fragments. Chromosomes I and II each release two telomeric restriction fragments (C, I, L, and M). Chromosome III lacks NotI restriction sites; NotI digests of genomic DNA of the indicated strains were separated by PFGE, and chromosomal end-to-end fusions were detected by Southern blotting with a telomere probe (arrows indicate positions of resolved telomere fusions). Nitrogen-starved *taz1d* used as positive control for fusions.

(B) PFGE analysis reveals that Xlf1.AA promotes NHEJ-mediated telomeric fusions in cycling cells in *taz1d ctp1d* background.

(C) Xlf1 overexpression is sufficient to promote telomeric fusions in *taz1d* cycling cells, and *xlf1.AA* mutation increases the amount of these fusions. Ligation of chromosome ends in *taz1d* strains overexpressing *xlf1* mutants from the *nmt41* promoter was analyzed by PFGE. *taz1*$^+$ control strains contain the same amount of DNA, though the signal from the telomeric probe is weaker as a result of telomere elongation in *taz1d*.

(D) Assaying for NHEJ and HR activities in parallel, using hygromycin resistance and leu1 integration of linearized plasmid fragments. Data are reported as the mean ± 95% confidence interval.

See also Figure S3C.

in favor of end-joining, either by promoting NHEJ before DSB becomes subject to HR or by separately inhibiting HR. These observations imply that *xlf1* is an important regulator in the balance of HR versus NHEJ during the cell cycle.

Xlf1.AA Affects Both HR and NHEJ of Linearized Plasmid DNA

The observation that chromosome end ligations (*taz1d* cells) in the *xlf1.AA* phosphomutant require inactivation of HR indicates crosstalk between NHEJ and HR. To verify if a similar effect could be identified in the processing of plasmid DNA ends by the two DSB repair pathways, we designed an assay in which the levels of HR and NHEJ can be assessed in parallel. Cells were electroporated with two different linearized plasmids. One plasmid, containing WT *leu1*$^+$ but no ARS, could be integrated at the *leu1-32* locus via an HR-dependent mechanism, giving rise to Leu+ colonies. The second plasmid, containing the antibiotic resistance gene *hph*^{R} and an *S. pombe* ARS, could be recircularized by NHEJ and stably maintained, giving rise to hygromycin-resistant colonies. Hygromycin selection was applied after 2 hr. Cells were transformed in parallel; the frequency of leu1$^+$ colonies and hygromycin-resistant colonies were determined in relation to an uncut plasmid control as a measure of HR and NHEJ activity, respectively. Deletion of *xlf1*$^+$ decreased plasmid religation ~50 fold (Figure 3D), whereas deletion of *ctp1*$^+$ decreased plasmid integration ~50 fold, indicative of inactivation of NHEJ and HR, respectively. Deletion of *xlf1*$^+$ or *lig4*$^+$ also caused a small but reproducible decrease in HR-dependent plasmid integration. Plasmid religation was increased in *xlf1.AA* mutant cells, though less than in the assay used in Figure 3D, presumably because of the much-reduced time available for religation with antibiotic rather than auxotrophic selection. Unexpectedly, plasmid integration was reduced by two-thirds in *xlf1.AA* (endogenous promoter), suggesting

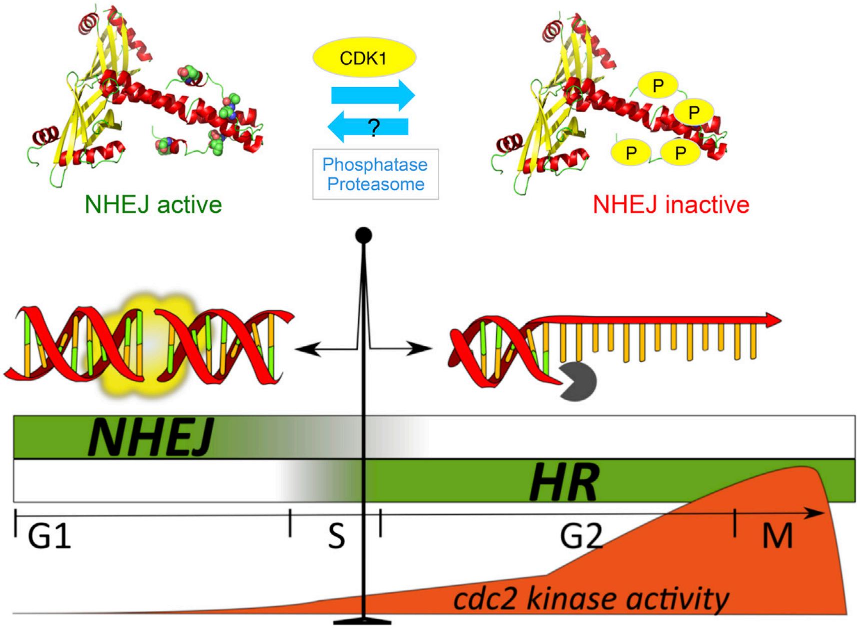

Figure 4. Model of Cdk1 Regulation of the Balance between NHEJ and HR

The activity of CDK1 is a major determinant of DSB repair pathway choice. When CDK1 is low, non-phosphorylated Xlf1 exists in the cell and NHEJ is fully active. As CDK1 activity rises, phosphorylation of Xlf1 increases, leading to the inactivation of NHEJ. The phosphorylation of CDK1 targets in the HR pathway leads to the activation of HR.

that disabling the phosphorylation of Xlf1 leads to inhibition of HR. In addition, while deletion of *ctp1*$^+$ by itself had no significant impact on NHEJ, deletion of *ctp1*$^+$ in an *xlf1.AA* mutant led to a much larger increase in plasmid religation than in HR-competent *xlf1.AA*. Together, these observations provide further evidence that phospho-null *xlf1.AA* affects the levels of HR.

Cell cycle regulation of DSB repair pathway selection by Cdk1 was first established with the discovery that DSB resection requires Cdk1 activity (Aylon et al., 2004; Ira et al., 2004). However, while Cdk1 has been shown to control the function of several HR factors, a reciprocal regulation of NHEJ by Cdk1 has not been reported. While budding yeast Ku70 and Ku80 contain several potential Cdk1 phosphorylation sites, their mutation does not affect NHEJ activity (Zhang et al., 2009). Moreover, the dependence of resection for Cdk1 activity can be overcome by the deletion of Ku, suggesting an indirect control over NHEJ by modulation of HR (Clerici et al., 2008). Nej1, the budding yeast XLF homolog, was discovered as a factor downregulating NHEJ in diploid cells (Frank-Vaillant and Marcand, 2001; Kegel et al., 2001; Valencia et al., 2001). Lif1, the *S. cerevisiae* XRCC4 homolog, has been found to be phosphorylated by Cdk1, but this phosphorylation has little effect on the levels of classical NHEJ and, instead, affects a Sae2^{ctp1}-dependent resection-mediated imprecise joining pathway (Matsuzaki et al., 2012). Our study shows that Cdk1 phosphorylation of a core NHEJ factor directly regulates classical NHEJ during the cell cycle. We show that Xlf1 becomes phosphorylated by Cdc2^{Cdk1} on T180 and S192 and that the levels of phosphorylation increase through the cell cycle as Cdc2^{Cdk1} activity increases. These results establish that phosphorylation of Xlf1 has an inhibitory effect, as there is a reduction in the levels of religation of linearized DNA with the phosphomimic *xlf1.EE* but an increase with the phospho-null *xlf1.AA* mutant. Together, these observations allow us to propose a model in which Xlf1 functions as a cellular switch for NHEJ (Figure 4), with nonphosphorylated Xlf1 representing the on state (NHEJ active) and phosphorylated Xlf1 representing the off state (NHEJ inactive). The NHEJ repair pathway is fully active in G_1, but as cells advance in the cell cycle, Cdc2^{Cdk1} levels rise and Xlf1 becomes increasingly phosphorylated, leading to inactivation of end-joining. Although this report establishes that Xlf1 is an important NHEJ regulator, further studies are required to establish how Cdk1 phosphorylation of this factor restrains NHEJ in cycling cells and whether this key regulatory function is also conserved in other eukaryotic organisms.

EXPERIMENTAL PROCEDURES

Standard methods used for strain construction, western blotting, and microscopy are detailed in the Supplemental Experimental Procedures. Strains used are listed in Table S1.

Phosphatase Treatment of *nmt41*-GFP-Tagged Xlf1

A total of 10^9 log-phase cells grown without thiamine was resuspended in lysis buffer (50 mM Na phosphate [pH 7], 150 mM NaCl, 50 mM NaF, 10 mM EDTA, 10% glycerol, 0.5% NP40, Roche protease inhibitor) and broken with glass beads. Cleared lysate containing 15 μg total protein was incubated for 2 hr at 4°C with 15 μl GFP-Trap A magnetic beads (Chromotek) per immunoprecipitation. Beads were washed with lysis buffer, washed three times in PMP buffer (New England Biolabs; NEB) plus $MnCl_2$, resuspended in 100 μl, divided into two, and either mock treated or incubated with 800 units of lambda phosphatase (NEB) at 30°C for 30 min. Beads were resuspended in Laemmli buffer and boiled. Samples were separated by SDS-PAGE on 12% gels containing 25 μM Phos-tag (AAL-107 Wako) and 50 μM $MnCl_2$. Prior to transfer onto polyvinylidene fluoride, gels were incubated for 10 min in transfer buffer with 1 mM EDTA and then without EDTA. GFP-Xlf1 was detected using anti-GFP antibody (Invitrogen, 1:2,500 dilution).

In Vitro Cdc2^{Cdk1} Kinase Assay

Active Cdc2 enzyme was isolated from a lysate of 5×10^8 WT *S. pombe* cells resuspended in 400 μl of HB buffer (25 mM Tris [pH 7.5], 15 mM EGTA, 15 mM $MgCl_2$, 0.1% NP40, protease inhibitor cocktail) broken using glass beads. Per kinase reaction, cell lysate containing 1 mg total cellular protein was mixed with 40 μl of p13suc1 agarose conjugate (Millipore), incubated with rotation at 4°C for 3 hr, washed three times with HB buffer, and then washed once with kinase buffer (10 mM HEPES [pH 7.5], 75 mM KCl, 5 mM $MgCl_2$, 1 mM dithiothreitol). For each kinase assay reaction, beads were then mixed in a total volume of 15 μl containing 1.5 μg purified protein (either recombinant 6His-Xlf1 or histone H1), 20 μM ATP, and 5μCi γ-^{32}P-ATP, all diluted in kinase buffer. The same method was used with Cdk1 (NEB). The kinase reaction was allowed to proceed for 10 min at room temperature and then was stopped by adding SDS-PAGE loading buffer and heated to 90°C for 5 min. Protein

was separated on 15% gels and subjected to Coomassie staining and autoradiography.

SUPPLEMENTAL INFORMATION

Supplemental Information includes Supplemental Experimental Procedures, three figures, and one table and can be found with this article online at http://dx.doi.org/10.1016/j.celrep.2014.11.044.

AUTHOR CONTRIBUTIONS

P.H. and A.J.D. designed the key experiments and wrote the manuscript with advice from M.G.F. and C.C.R. H.W. designed and carried out hygromycin plasmid assays and spore survivals and made *chk1* and *rad52* strains. Experimental work using *Taz1d* was designed by M.G.F. and C.C.R. C.C.R carried out and made strains needed for these *taz1* experiments. P.H. carried out all other experimental work and strain production.

ACKNOWLEDGMENTS

Work in the A.J.D. laboratory was supported by grants from Cancer Research UK (C1470/A12430) and the Biotechnology and Biological Sciences Research Council (BB/M004236/1) and by a centre grant from the Medical Research Council (MRC; G0801130). The M.G.F. laboratory was supported by the Portuguese Fundação para a Ciência e Técnologia (FCT; PTDC/SAU-OBD/66438/2006 and PTDC/BIA-BCM/099367/2008). C.C.R. was supported by an FCT postdoctoral fellowship. H.W. was supported by an MRC-Doctoral Training Account PhD studentship. The authors thank Drs. E. Hartsuiker, V. Garcia, and A. Watson for technical assistance and Prof. A. Carr and Dr. J. Murray for useful discussions. M.G.F. is a Howard Hughes Medical Institute International Early Career Scientist. Funding for open access charges was provided by Research Councils UK.

Received: June 23, 2014
Revised: October 28, 2014
Accepted: November 25, 2014
Published: December 18, 2014

REFERENCES

Almeida, H., and Godinho Ferreira, M. (2013). Spontaneous telomere to telomere fusions occur in unperturbed fission yeast cells. Nucleic Acids Res. *41*, 3056–3067.

Aylon, Y., Liefshitz, B., and Kupiec, M. (2004). The CDK regulates repair of double-strand breaks by homologous recombination during the cell cycle. EMBO J. *23*, 4868–4875.

Cavero, S., Chahwan, C., and Russell, P. (2007). Xlf1 is required for DNA repair by nonhomologous end joining in Schizosaccharomyces pombe. Genetics *175*, 963–967.

Chiruvella, K.K., Liang, Z., and Wilson, T.E. (2013). Repair of double-strand breaks by end joining. Cold Spring Harb. Perspect. Biol. *5*, a012757.

Clerici, M., Mantiero, D., Guerini, I., Lucchini, G., and Longhese, M.P. (2008). The Yku70-Yku80 complex contributes to regulate double-strand break processing and checkpoint activation during the cell cycle. EMBO Rep. *9*, 810–818.

Dischinger, S., Krapp, A., Xie, L., Paulson, J.R., and Simanis, V. (2008). Chemical genetic analysis of the regulatory role of Cdc2p in the S. pombe septation initiation network. J. Cell Sci. *121*, 843–853.

Ferreira, M.G., and Cooper, J.P. (2004). Two modes of DNA double-strand break repair are reciprocally regulated through the fission yeast cell cycle. Genes Dev. *18*, 2249–2254.

Ferretti, L.P., Lafranchi, L., and Sartori, A.A. (2013). Controlling DNA-end resection: a new task for CDKs. Front. Genet. *4*, 99.

Frank-Vaillant, M., and Marcand, S. (2001). NHEJ regulation by mating type is exercised through a novel protein, Lif2p, essential to the ligase IV pathway. Genes Dev. *15*, 3005–3012.

Hentges, P., Ahnesorg, P., Pitcher, R.S., Bruce, C.K., Kysela, B., Green, A.J., Bianchi, J., Wilson, T.E., Jackson, S.P., and Doherty, A.J. (2006). Evolutionary and functional conservation of the DNA non-homologous end-joining protein, XLF/Cernunnos. J. Biol. Chem. *281*, 37517–37526.

Huertas, P., Cortés-Ledesma, F., Sartori, A.A., Aguilera, A., and Jackson, S.P. (2008). CDK targets Sae2 to control DNA-end resection and homologous recombination. Nature *455*, 689–692.

Ira, G., Pellicioli, A., Balijja, A., Wang, X., Fiorani, S., Carotenuto, W., Liberi, G., Bressan, D., Wan, L., Hollingsworth, N.M., et al. (2004). DNA end resection, homologous recombination and DNA damage checkpoint activation require CDK1. Nature *431*, 1011–1017.

Kegel, A., Sjöstrand, J.O., and Aström, S.U. (2001). Nej1p, a cell type-specific regulator of nonhomologous end joining in yeast. Curr. Biol. *11*, 1611–1617.

Kinoshita, E., Kinoshita-Kikuta, E., Takiyama, K., and Koike, T. (2006). Phosphate-binding tag, a new tool to visualize phosphorylated proteins. Mol. Cell. Proteomics *5*, 749–757.

Limbo, O., Chahwan, C., Yamada, Y., de Bruin, R.A.M., Wittenberg, C., and Russell, P. (2007). Ctp1 is a cell-cycle-regulated protein that functions with Mre11 complex to control double-strand break repair by homologous recombination. Mol. Cell *28*, 134–146.

Manolis, K.G., Nimmo, E.R., Hartsuiker, E., Carr, A.M., Jeggo, P.A., and Allshire, R.C. (2001). Novel functional requirements for non-homologous DNA end joining in Schizosaccharomyces pombe. EMBO J. *20*, 210–221.

Matsuzaki, K., Terasawa, M., Iwasaki, D., Higashide, M., and Shinohara, M. (2012). Cyclin-dependent kinase-dependent phosphorylation of Lif1 and Sae2 controls imprecise nonhomologous end joining accompanied by double-strand break resection. Genes Cells. *17*, 473–493.

Moreno, S., Hayles, J., and Nurse, P. (1989). Regulation of p34cdc2 protein kinase during mitosis. Cell *58*, 361–372.

Symington, L.S., and Gautier, J. (2011). Double-strand break end resection and repair pathway choice. Annu. Rev. Genet. *45*, 247–271.

Valencia, M., Bentele, M., Vaze, M.B., Herrmann, G., Kraus, E., Lee, S.E., Schär, P., and Haber, J.E. (2001). NEJ1 controls non-homologous end joining in Saccharomyces cerevisiae. Nature *414*, 666–669.

Walworth, N.C., and Bernards, R. (1996). rad-dependent response of the chk1-encoded protein kinase at the DNA damage checkpoint. Science *271*, 353–356.

Zhang, Y., Shim, E.Y., Davis, M., and Lee, S.E. (2009). Regulation of repair choice: Cdk1 suppresses recruitment of end joining factors at DNA breaks. DNA Repair (Amst.) *8*, 1235–1241.

Cell Reports, Volume *9*

Supplemental Information

Cdk1 Restrains NHEJ through Phosphorylation of XRCC4-like Factor Xlf1

Pierre Hentges, Helen Waller, Clara C. Reis, Miguel Godinho Ferreira, and Aidan J. Doherty

Supplemental Table 1: strains used

Name	genotype
PH111	xlf1:: natMX-nmt41-GFP-xlf1::loxP/M
PH113	xlf1:: natMX-nmt41-GFP-xlf1.T180A.S192A::loxP/M
PH369	cdc2.F84G xlf1:: natMX-nmt41-GFP-xlf1::loxP/M
PH371	cdc2.F84G xlf1:: natMX-nmt41-GFP-xlf1.T180A.S192A::loxP/M
PH365	cdc10.M17 xlf1:: natMX-nmt41-GFP-xlf1::loxP/M
PH360	cdc10.M17 xlf1:: natMX-nmt41-GFP-xlf1.T180A.S192A::loxP/M
PH001	ura4D-18 leu1-32 nmt41-xlf1::natMX
PH095	ade6-704 leu1-32 ura4-D18 xlf1::xlf1::loxP/M
PH097	ade6-704 leu1-32 ura4-D18 xlf1::xlf1. T180A.S192A::loxP/M
PH099	ade6-704 leu1-32 ura4-D18 xlf1::xlf1. T180E.S192E::loxP/M
MGF490	ade6-704 leu1-32 ura4-D18 taz1::natMX xlf1::ura4::loxP/M
MGF491	ade6-704 leu1-32 ura4-D18 xlf1::xlf1::loxP/M taz1::kan
MGF492	ade6-704 leu1-32 ura4-D18 xlf1.T180A.S192A::loxP/M taz1::kan
MGF493	ade6-704 leu1-32 ura4-D18 xlf1.T180E.S192E::loxP/M taz1::kan
MGF633	ade6-704 leu1-32 ura4-D18 xlf1.T180E.S192E::loxP/M xlf1EE ctp1::KanR taz1::URA4+
MGF669	leu1-32 ura4-D18 xlf1.T180A.S192A::loxP/M ctp1::KanR taz1::URA4+
MGF672	ura4D-18 leu1-32 nmt41-xlf1::natMX taz1::URA4+
MGF673	ura4D-18 leu1-32 nmt41-xlf1.AA::natMX taz1::URA4+
MGF1776	ade6-704 leu1-32 ura4-D18 xlf1.T180A.S192A::loxP/M taz1::kan lig4::natMX ctp1::hphMX

Table of the strains used and their genotypes. Related to Experimental Procedures.

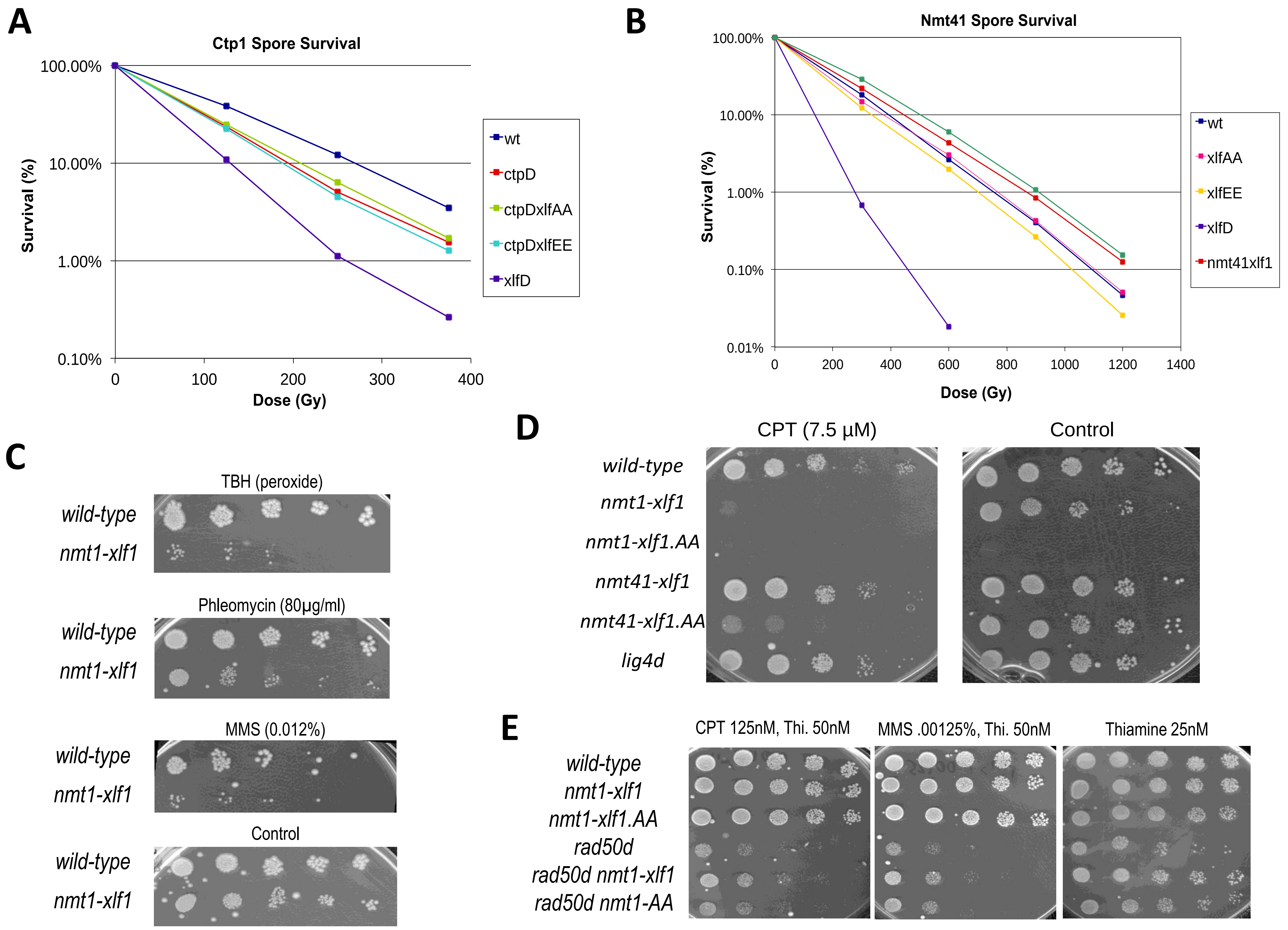
A
Ctp1 Spore Survival
100.00%
10.00%
1.00%
0.10%
Survival (%)
0
100
200
300
400
Dose (Gy)
wt
ctpD
ctpDxlfAA
ctpDxlfEE
xlfD
B
Nmt41 Spore Survival
100.00%
10.00%
1.00%
0.10%
0.01%
Survival (%)
0
200
400
600
800
1000
1200
1400
Dose (Gy)
wt
xlfAA
xlfEE
xlfD
nmt41xlf1
C
TBH (peroxide)
wild-type
nmt1-xlf1
Phleomycin (80µg/ml)
wild-type
nmt1-xlf1
MMS (0.012%)
wild-type
nmt1-xlf1
Control
wild-type
nmt1-xlf1
D
CPT (7.5 µM)
Control
wild-type
nmt1-xlf1
nmt1-xlf1.AA
nmt41-xlf1
nmt41-xlf1.AA
lig4d
E
CPT 125nM, Thi. 50nM
MMS .00125%, Thi. 50nM
Thiamine 25nM
wild-type
nmt1-xlf1
nmt1-xlf1.AA
rad50d
rad50d nmt1-xlf1
rad50d nmt1-AA

Figure S1

Supplemental Figure Legends

Figure S1 related to Figure 1

A. Spore survival of spores deleted for *ctp1*

B. Spore survival of *xlf1* point mutants

C. Spot tests with DNA damage treatment of strains overexpressing *xlf1* from medium-level *nmt41*

D. Spot tests with DNA damage treatment of *xlf1* mutants overexpressing from medium-level *nmt41* and high-level *nmt1*

E. Spot tests with DNA damage treatment of *xlf1* overexpression in *rad50* deletion background

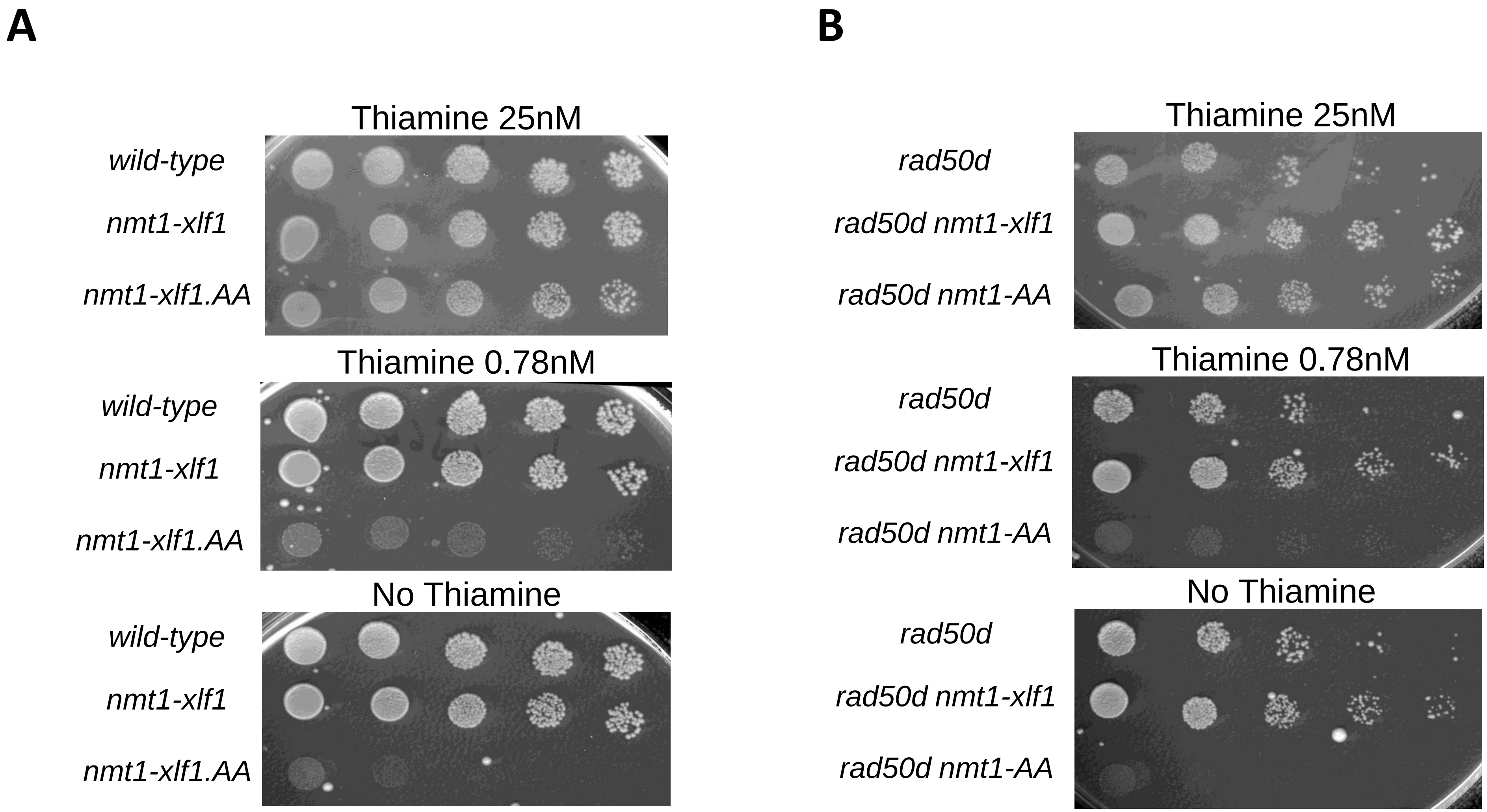
A
Thiamine 25nM
wild-type
nmt1-xlf1
nmt1-xlf1.AA
Thiamine 0.78nM
wild-type
nmt1-xlf1
nmt1-xlf1.AA
No Thiamine
wild-type
nmt1-xlf1
nmt1-xlf1.AA
B
Thiamine 25nM
rad50d
rad50d nmt1-xlf1
rad50d nmt1-AA
Thiamine 0.78nM
rad50d
rad50d nmt1-xlf1
rad50d nmt1-AA
No Thiamine
rad50d
rad50d nmt1-xlf1
rad50d nmt1-AA

Figure S2

Figure S2 related to Figure 2

A. Spot test with nmt1 high-level overexpression of *xlf1* mutants.

B. Spot test with nmt1 high-level overexpression of *xlf1* mutants in *rad50d* background.

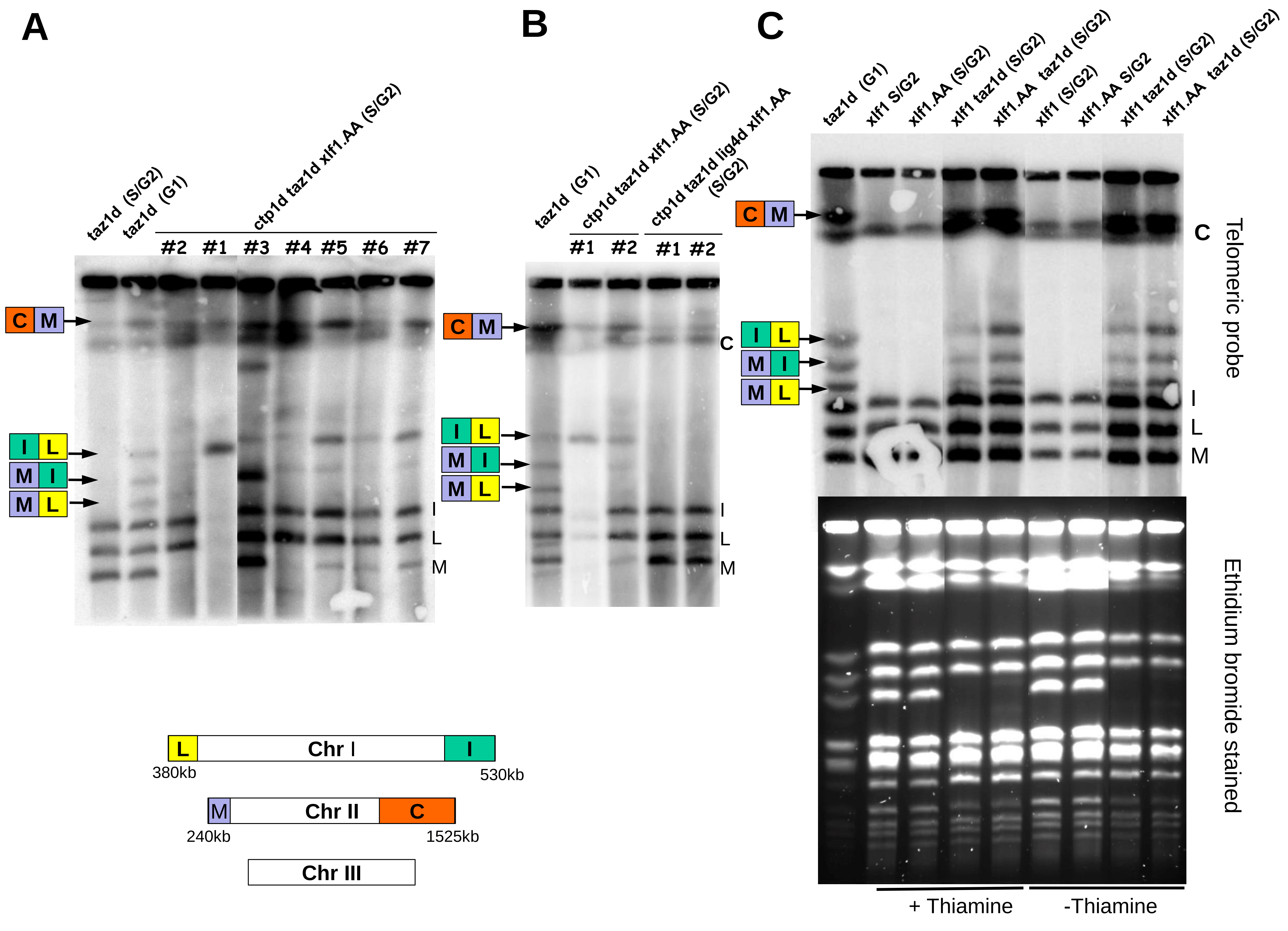
A
taz1d (S/G2)
taz1d (G1)
ctp1d taz1d xlf1.AA (S/G2)
#2 #1 #3 #4 #5 #6 #7
C M
I L
M I
M L
I
L
M
B
taz1d (G1)
ctp1d taz1d xlf1.AA (S/G2)
ctp1d taz1d lig4d xlf1.AA (S/G2)
#1 #2 #1 #2
C
C
taz1d (G1)
xlf1 S/G2
xlf1.AA (S/G2)
xlf1 taz1d (S/G2)
xlf1.AA taz1d (S/G2)
xlf1 (S/G2)
xlf1.AA S/G2
xlf1 taz1d (S/G2)
xlf1.AA taz1d (S/G2)
Telomeric probe
Ethidium bromide stained
+ Thiamine
-Thiamine
L
Chr I
I
380kb
530kb
M
Chr II
C
240kb
1525kb
Chr III

Figure S3

Figure S3 related to Figure 3

A. 7 independent clones were isolated by deleting *taz1* in *ctp1d xlf1.AA* cells through integration of a *kanMX* antibiotic resistance cassette. *xlf1.AA* is expressed at endogenous levels.

Diagram of telomeric NotI restriction fragments. Chromosomes I and II each release two telomeric restriction fragments (C, I, L and M). Chromosome III lacks NotI restriction sites; NotI digests of genomic DNA of the indicated strains were separated by PFGE and chromosomal end-to-end fusions were detected by Southern blot using a telomere probe (arrows indicate the positions of the resolved telomere fusions). Nitrogen starved *taz1d* was used as positive control for fusions.

B. NHEJ-mediated telomeric fusions in cycling cells of two independent clones of *ctp1d taz1d xlf1.AA* and two independent clones of *ctp1d taz1d lig4d xlf1.AA*. PFGE analysis and Southern blot using a using a telomere probe.

C. Southern blot (top) and ethidium bromide stained agarose gel (bottom) of PFGE shown in Fig 3. Decreases in the signal intensity from the telomere probe are not due to less DNA being loaded. Wild-type *xlf1* and *xlf1.AA* are expressed from the medium-strength thiamine-derepressible *nmt41* promoter.

Experimental Procedures

Genetic and Cell Studies

Media and standard genetic techniques were as described previously (Moreno et al., 1991). Spot tests and spore survival assays were carried out as in (Hentges et al., 2006) *xlf1* phosphorylation mutant strains were created by integrating DNA fragments containing T180A.S192A and T180E.S192E at the *xlf1* locus using a cre-lox method described in (Watson et al., 2008). *nmt41, nmt1* and *nmt41-GFP* strains were derived from this by integrating the corresponding cassettes described in (Van Driessche et al., 2005). Nat deletion strains were constructed as in (Hentges et al., 2005). For the *taz1d* assays, strains were created by integrating a kanMX cassette into the relevant background at the *taz1* locus while assays was carried out as described previously (Reis et al., 2012).

To determine the formation of Rad52 foci after IR, *nmt1-xlf1 rad52-GFP* and *nmt1-xlf1.AA rad52-GFP* cells were grown to log-phase in YNB without thiamine. A population of early G2 cells was isolated using centrifugation through a lactose gradient. Cells were treated with 50Gy gamma irradiation, attached to a concanavalin A-coated chamber. The presence of Rad52 foci was monitored over 4 hours.

DSB Plasmid Repair Assays

The plasmid religation assay using leucine selection (fig 2.a) was carried out as described previously (Manolis et al., 2001). The plasmid relegation assay using hygromycin selection (fig 2.b) was carried out in the same manner except that plasmid pRL1 bearing a hphMX marker was used, and that plasmid transformation was followed by a 2 hour incubation step before hygromycin selection was applied by plating cells on hygromcyin-containing media. The plasmid repair assay measuring HR and NHEJ in parallel (fig. 3.d) used plasmids pRL1 (hygromycin) for end-joining and pJK148 (leu1 marker) for chromosomal integration at *leu1*. 2x10^8 mid-log phase cells were washed and incubated in 4ml DTT buffer for 15min at 30°C. Cells were washed twice with 2ml 1M sorbitol, resuspended in a total volume of 100μl by adding 70μl 1M sorbitol and then divided in two. For the NHEJ electroporations, 200ng uncut pAL19 and 600ng EcoRV and PvuII digested pRL1 plasmid. For the HR electroporations add 600ng uncut pRL1 and 400ng NdeI digested pJK148

plasmid DNA. Cells were then transferred to pre chilled electroporation cuvettes (0.2cm), electroporated at 1500V, 200Ω, 25μF. 950μL ice cold YNB with 1M sorbitol was added immediately and cells were incubated at 30°C for 2 hours. Cells were then plated on selective plates (YNB +ura +ade -leu, YEA +hyg) and incubated at 30°C for 4-5 days. NHEJ efficiency was calculated by dividing the number of hygromycin resistant colonies by the number of leucine positive colonies. HR efficiency was calculated by dividing leu+ colonies by the number of hygromycin resistant colonies. Values were normalised to wt = 100% for both repair pathways.

Protein methods

To separate and detect phosphorylated Xlf1 using the Phos-tag, samples were separated by SDS-PAGE on 12% gels containing 25μM Phos-tag (AAL-107 Wako) and 50μM $MnCl_2$. Prior to transfer onto PVDF, gels were incubated for 10min in transfer buffer with 1mM EDTA, then buffer without EDTA, followed by standard Western blotting. GFP-Xlf1 was detected using anti-GFP antibody from Invitrogen (1:2500 dilution).

For the experiment with *cdc10.M17* ts cells, cultures were grown to log-phase in EMM2 without thiamine at 25°C, blocked by growth at 36.5°C for one generation time, and released by switching temperature to 25°C. Whole cell lysates were prepared using the TCA method (Watson et al., 2008) and analysed by Western blotting with Phos-tag as described above.

Chk1 phosphorylation was analysed using lysates prepared using the TCA method, and detection of Chk1-HA using monoclonal anti-HA antibody (diluted 1:2000; Santa Cruz Biotechnology).

Recombinant Xlf1 protein with the mutations T180A, S192A, or T180A.S192A was prepared by site-directed mutagenesis of xlf1 on pET28a, followed by expression and purification as described previously (Hentges et al., 2006).

References

Van Driessche, B., Tafforeau, L., Hentges, P., Carr, A.M., and Vandenhaute, J. (2005). Additional vectors for PCR-based gene tagging in Saccharomyces cerevisiae and Schizosaccharomyces pombe using nourseothricin resistance. Yeast Chichester Engl. *22*, 1061–1068.

Hentges, P., Van Driessche, B., Tafforeau, L., Vandenhaute, J., and Carr, A.M. (2005). Three novel antibiotic marker cassettes for gene disruption and marker switching in Schizosaccharomyces pombe. Yeast Chichester Engl. *22*, 1013–1019.

Hentges, P., Ahnesorg, P., Pitcher, R.S., Bruce, C.K., Kysela, B., Green, A.J., Bianchi, J., Wilson, T.E., Jackson, S.P., and Doherty, A.J. (2006). Evolutionary and functional conservation of the DNA non-homologous end-joining protein, XLF/Cernunnos. J. Biol. Chem. *281*, 37517–37526.

Manolis, K.G., Nimmo, E.R., Hartsuiker, E., Carr, A.M., Jeggo, P.A., and Allshire, R.C. (2001). Novel functional requirements for non-homologous DNA end joining in Schizosaccharomyces pombe. EMBO J. *20*, 210–221.

Moreno, S., Klar, A., and Nurse, P. (1991). Molecular genetic analysis of fission yeast Schizosaccharomyces pombe. Methods Enzymol. *194*, 795–823.

Reis, C.C., Batista, S., and Ferreira, M.G. (2012). The fission yeast MRN complex tethers dysfunctional telomeres for NHEJ repair. EMBO J. *31*, 4576–4586.

Watson, A.T., Garcia, V., Bone, N., Carr, A.M., and Armstrong, J. (2008). Gene tagging and gene replacement using recombinase-mediated cassette exchange in Schizosaccharomyces pombe. Gene *407*, 63–74.
